# Low Energy Diets for Obesity and CKD (SLOW-CKD Randomized Feasibility Study)

**DOI:** 10.1016/j.ekir.2025.04.021

**Published:** 2025-04-21

**Authors:** Marguerite M. Conley, Hannah L. Mayr, Kirsten S. Hepburn, Justin J. Holland, David W. Mudge, Tammy J. Tonges, Richard S. Modderman, Sally A. Gerzina, David W. Johnson, Andrea K. Viecelli, Helen L. MacLaughlin

**Affiliations:** 1 School of Exercise and Nutrition Sciences, Queensland University of Technology, Brisbane, Queensland, Australia; 2 Department of Nutrition and Dietetics, Princess Alexandra Hospital, Brisbane, Queensland, Australia; 3 Department of Kidney and Transplant Services, Princess Alexandra Hospital, Brisbane, Queensland, Australia; 4 Faculty of Medicine, The University of Queensland, Brisbane, Queensland, Australia; 5 Centre for Functioning and Health Research, Metro South Hospital and Health Service, Brisbane, Queensland, Australia; 6 Kidney Health Service, Royal Brisbane and Women’s Hospital, Brisbane, Queensland, Australia; 7 Australasian Kidney Trials Network, University of Queensland, Brisbane, Queensland, Australia; 8 Redland Hospital, Brisbane, Queensland, Australia; 9 Consumer Co-researcher, Brisbane, Queensland, Australia; 10 Flinders Health and Medical Research Institute, College of Medicine and Public Health, Flinders University, Bedford Park South Australia, Australia; 11 Department of Physiotherapy, Sunshine Coast University Hospital, Birtinya, Queensland, Australia; 12 Nutrition Research Collaborative, Dietetics and Foodservices, Royal Brisbane and Women’s Hospital, Brisbane, Queensland, Australia

**Keywords:** chronic kidney disease, low energy diet, meal replacement, obesity, weight loss, weight management

## Abstract

**Introduction:**

Low energy diets (LEDs) may slow disease progression; however, their effects are under researched in chronic kidney disease (CKD). This study evaluated the safety and feasibility of an LED weight management program in adults with obesity and CKD.

**Methods:**

This multicenter 6-month randomized controlled trial (RCT) involved adults with CKD Kidney Disease: Improving Global Outcomes stages G1 to G3b, obesity, and proteinuria, randomized 1:1 into 2 groups. The LED group followed a 3-month 800 to 900 kcal/d LED, with dietitian support, then a 3-month weight maintenance phase with exercise and healthy eating support. The usual care (UC) group received standard clinic weight loss support. Primary outcomes were safety (serious adverse events [SAEs]) and feasibility (≥2 of recruitment rate ≥ 25%, LED group retention rate ≥ 75%, and ≥ 30% of LED group achieving ≥ 10 kg weight loss at 3 months). Secondary outcomes included changes in anthropometry, clinical measures, patient-reported outcomes, and participant experiences.

**Results:**

Forty-nine participants (median age 51 years, 57% male) consented. SAEs were low and comparable as follows: 2 in the LED group (hypoglycemia and acute kidney injury) and 2 in the UC group (hypoglycemia), all requiring hospitalization. Feasibility was met for recruitment (46%) and weight loss (46% achieved ≥ 10 kg loss) but not for retention (67% retained). At 6 months, median (IQR) weight change was −9.0 kg (−12 to −7) in the LED group and 0 kg (−4 to 2) in the UC group (*P* < 0.001).

**Conclusion:**

LEDs under professional guidance are safe and feasible for weight loss in adults with obesity and CKD Kidney Disease: Improving Global Outcomes stages G1 to G3b. A definitive RCT to assess their effects on clinical outcomes and CKD progression is warranted.

## Graphical abstract

**Figure:**
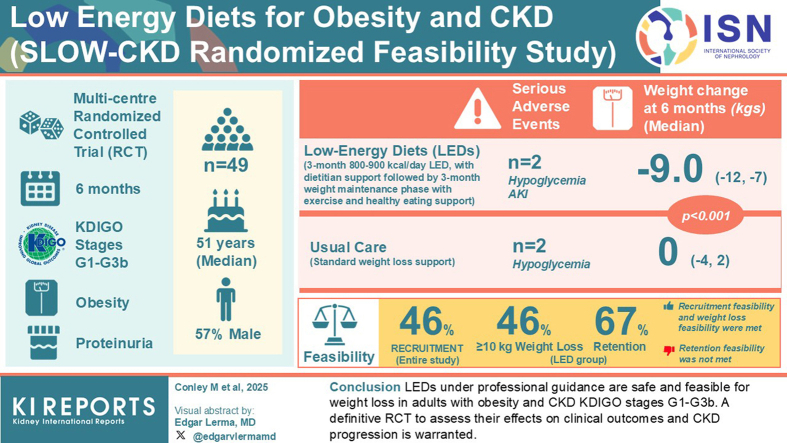

See Commentary on Page 2088

Obesity is a modifiable risk factor for CKD development^1^ and progression,^2^ and currently affects 30% to 50% of people with CKD.^3^^,^^4^ With excess weight in this population now contributing to a quarter of all disability-adjusted life years worldwide,^5^ treatment of obesity in CKD is a priority. International practice guidelines recommend weight reduction for managing excess body weight in people living with CKD.^6^^,^^7^ However, specific management guidance for obesity in CKD is not well-established because of insufficient evidence on how modifying obesity risk factors impacts disease progression.^8^^,^^9^

Bariatric surgery is currently the most effective treatment pathway for sustained weight loss in severe obesity^10^ and is a viable option for people with CKD. A sustained average weight reduction of 24% to 26% is reported.^11^^,^^12^ However, not everyone wishes to have surgery, there is a risk of complications and nutritional deficiencies, and access remains limited. Emerging incretin-based pharmacotherapies, either alone or combined with other agents as dual or triple agonists, have resulted in average sustained weight losses of up to 10% in adults with type 2 diabetes and up to 20% in those without.^13^^,^^14^ These treatments have also shown reductions in risks of cardiovascular and kidney disease, and all-cause mortality.^14^, ^15^, ^16^ However, these medications have side effects, recurring high costs,^17^ and global shortages are impacting continuity of care.^18^ Furthermore, pharmacotherapy is not considered a standalone approach to weight management and greater weight loss has been achieved when combining medication with diet changes and exercise.^14^ Therefore, there remains a need for accessible, safe, cost-effective, and sustainable nonsurgical interventions to address the rising global burden of obesity and its associated health consequences (cardiovascular disease, morbidity, and mortality) in people with CKD.

Very LEDs (VLED, < 800 kcal/d [3.0 MJ/d]), and LEDs (800–1200 kcal/d [3.4–5.0 MJ/d])^19^ can result in substantial weight losses (10–15 kg), lower cardiovascular disease risk and put diabetes into remission.^20^^,^^21^ However, their use in adults with CKD is limited, with no published RCTs on their safety or efficacy in this population.^8^ Theoretically, VLEDs should lead to greater weight loss than LEDs because of greater energy restriction. However, studies show that although VLEDs result in more initial weight loss, the overall weight loss between VLEDs and LEDs is similar after 12 months.^19^^,^^22^ In addition, LEDs might support better adherence, greater preservation in muscle mass and lower participant risk, making them a more favorable option.^19^^,^^22^ A recent mixed methods study involving adults with CKD found a 4-week LED to be acceptable and safe with high-self reported adherence rates.^23^ This research is encouraging and highlights the need for further investigation into the use of LEDs plus specialized support over the longer-term. Before undertaking a definitive trial, the safety and feasibility of LEDs should be tested in adults with CKD. Therefore, the primary aim of this study was to test the safety and feasibility (recruitment and retention rate, and weight loss) of an LED and weight maintenance intervention in individuals with obesity and CKD.

## Methods

### Trial Design and Participants

This parallel, single-blinded, randomized controlled feasibility study was conducted from March 2022 to February 2024 across 3 public hospitals in Brisbane, Australia (Australian New Zealand Clinical Trials Register: ANZCTR 12622000069752). Participants included adults aged 18 to 75 years, with stages G1 to G3b CKD,^24^ obesity (body mass index ≥ 30 kg/m^2^), and persistent albuminuria (urinary albumin-to-creatinine ratio > 3mg/mmol). Exclusion criteria were as follows: (i) previous or planned bariatric surgery within the next 12 months; (ii) weight loss ≥ 5% within the previous 6 months; (iii) pregnancy or breastfeeding; (iv) kidney transplant recipient; (v) significant psychiatric or psychological disorder, including eating disorders; (vi) allergy or dietary intolerance or medical condition, whereby a formulated meal replacement or prepared commercial meals would be unsafe; (vii) deemed unsafe for an LED by a nephrologist; or (viii) > 1 episode of severe hyperkalemia (serum potassium ≥ 6.5 mmol/l) or acute kidney injury. The study was approved by the Royal Brisbane and Women’s Hospital (HREC/2021/QRBW/76570) and Queensland University of Technology (2021-4982-6543) Ethics Committees and adhered to the Declaration of Helsinki. The study protocol can be found on the Open Science Framework: https://osf.io/tcnru

### Recruitment and Randomization

Adults attending hospital nephrology outpatient clinics were screened for eligibility. Clearance from their nephrologist was obtained before inviting them to participate via phone, letter, or text. After providing written informed consent, participants completed a baseline assessment and were randomized to either the LED group or UC. The statistician generated a 1:1 randomization sequence with randomly permuted blocks of 10, stratified by hospital site and diabetes status, which was uploaded into the web-based research management system.^25^ Because of the nature of the intervention, participants, nephrologists, and clinicians delivering the intervention were aware of group assignment. Researchers undertaking data collection, laboratory staff, and the study statistician were blinded to group allocation.

### Study Procedures

All participants continued to attend their usual kidney clinic visits as scheduled, with treatment aiming for optimal blood pressure, lipid, and glycemic control using standard medical therapies according to national guidelines. Study visits were scheduled in person at baseline, 3 months, and 6 months.

#### Intervention

The intervention was developed by the research team, which included a consumer with lived experience of obesity, CKD, and LEDs, and was based on our previous work^23^ and current literature.^20^

#### LED Phase

Participants in the LED group followed a 3-month LED (∼800 to 900 kcal, ∼80 g protein/d), using commercial meal replacements (Optifast, Nestlé Health Science product ∼200 to 225 kcal each) and small low energy meals or snacks (∼150 to 250 kcal each) commercially available from supermarkets. They could choose either 4 meal replacements or 3 meal replacements plus 1 lowenergy meal or snack. Our preliminary study identified this flexible LED approach of using meal replacements and standardized snacks or small meals to accommodate various patient preferences.^23^ Participants were also encouraged to consume 1 piece of fruit, 2 cups of non-starchy vegetables or salads, and at least 2 liters of calorie-free fluids daily (if not on a fluid restriction). Optifast products, including shakes, soups, and bars, were provided, while participants purchased additional low energy meals/snacks, fruit, and vegetables. Participants received an information booklet detailing how to follow the LED, manage side effects and lists and photographs of suitable low energy meal and snacks items. Participants were instructed to refrain from consuming any food or fluids other than those provided or prescribed during this phase and encouraged to maintain their usual level of physical activity. To support adherence, the information booklet included an optional journal section where participants could record their adherence using provided checklists, note any side effects, daily reflections, challenges, and extra foods or fluids consumed. Participants were encouraged to discuss journal entries with the dietitian.

During the LED phase, participants were offered fortnightly contact with a dietitian (six 30–45-minute sessions), either in structured educational support groups via videoconference or 1-on-1 telephone calls. Sessions were tailored to individuals’ needs and included topics such as following an LED, awareness of hunger and satiety, eating habits, reasons for eating, and problem solving using a motivational interviewing approach. Additional dietitian support via phone, text, or email was available upon request or offered to those experiencing side effects or struggling with adherence. Participants could also join a virtual patient support group chat via WhatsApp to share experiences and receive peer support. The LED phase was a 3-month intervention for all participants regardless of weight loss achieved.

#### Weight-Loss Maintenance Phase

Following the LED phase, participants entered a 3-month weight-loss maintenance phase with support for diet and exercise. This phase focused on transitioning off the LED in a stepwise manner, pragmatically, and aligned with participant readiness. The aim was to maintain weight loss, build self-efficacy and establish routines for healthy eating^26^ and increased physical activity. Participants continued regular dietitian contact through monthly sessions (3 to four 30-minute sessions), either as structured group educational support via videoconference or individual telephone calls. Participants continued to have access to the WhatsApp group. Participants received a second information booklet with guidance on healthy eating, portion sizes, label reading, sample meal ideas or snacks, recipes, and strategies for being more active.^26^, ^27^, ^28^ If weight gain exceeded 2 kg, participants were encouraged to use meal replacements or low energy meals for 1 to 2 meals daily for 1 to 2 weeks.

During this phase, participants had access to fortnightly support from an exercise physiologist (EP) (6 individual videoconference sessions, 30–45 minutes) for the development of an individualized, graded home exercise program based on their self-identified activity goals. In line with exercise recommendations for CKD,^29^ the program included a combination of upper and lower body movements, cardiovascular exercises, and mobility exercises, aiming to increase strength, maintain or improve muscle mass, and enhance cardiovascular fitness. The EP encouraged participants to aim for 150 minutes of moderate-intensity exercise per week aligning with the Australian Physical Activity guidelines.^28^ The intensity and volume of exercise was tailored based on each participant’s medical history, disease state, goals, and identified barriers. The exercise program was entered into the PhysiTrak software or emailed to participants to complete at home at their own convenience. PhysiTrak software was used to monitor whether participants completed the prescribed exercise program. Additional support from the EP via telephone, text message, and videoconference was available to participants upon request or offered by the EP if prescribed exercise sessions outlined in PhysiTrak were not recorded as completed.

#### UC

Participants had the option of weight loss support through their usual kidney clinic, involving referral to a dietitian or weight loss program if available and participant agreed.

### Safety Management

Safety data were collected every 2 to 4 weeks for LED participants and at 3- and 6-month appointments for UC participants, using self-reports and electronic medical records. LED participants reported any side effects or medical concerns during dietitian contact and were encouraged to consult their general practitioner for medication and blood pressure monitoring. LED participants had a blood test 1 week after starting the LED to monitor serum electrolytes and estimated glomerular filtration rate (eGFR). Results were reviewed by a nephrologist who arranged for any necessary repeat testing or clinical interventions. LED participants with diabetes had their medications reviewed by a nephrologist or endocrinologist before starting the diet and received ongoing support as clinically required, from a diabetes nurse practitioner or endocrinologist for blood glucose management and medication adjustments throughout the study. An independent data safety committee, including a nephrologist, an EP, and dietitian, reviewed all SAEs and adverse events requiring medical intervention.

### Primary Outcomes

Primary outcomes included safety (based on study-related SAEs) and feasibility (recruitment rate, retention rate, and weight loss). Predefined safety and feasibility criteria are presented in Table 1. The intervention needed to be deemed safe and meet 2 out of 3 feasibility criteria to be considered suitable for progression to a definitive trial.

**Table 1:** Primary outcome measures of safety and feasibility for the SLOW-CKD feasibility study in adults with obesity and chronic kidney disease compared with prespecified criteria

Primary outcome	Prespecified criteria	Result	Criteria met
Safety	The proportion of study-related SAEs^a^ in both groups is similar	2 SAEs in each group.	Yes
Feasibility			
Recruitment rate	≥ 25 % of all eligible patients who can be contacted are recruited	46% (49/107) were recruited	Yes
Retention rate^b^	≥ 75% of recruited LED participants retained at 6 mos	67% (16/24) of LED and 88% (22/25) of UC participants were retained	No
Weight loss	≥ 30% of LED participants with least 10 kg weight loss at 3 mos	46% (11/24) of LED and 4% (1/25) of UC participants with ≥ 10 kg weight loss	Yes

SAE, serious adverse event; LED, low energy diet; UC, usual care.

Success was defined if the intervention was safe AND if 2 or more of the 3 feasibility criteria, namely recruitment, retention, and weight loss, were met. Feasibility criteria were developed by the research team, based on their clinical expertise, clinical judgement and previous work.^23^

a SAEs are defined as any possible study related adverse event that led to death, life-threatening adverse event, inpatient hospitalization or prolongation of existing hospitalization.

b Defined as completing the 6-month study visit.

### Secondary Outcomes

Secondary outcomes evaluated the clinical effectiveness and feasibility of measuring clinical parameters, biochemical markers, functional measures, and patient-reported outcomes in a weight loss trial. These assessments were conducted at baseline, 3 months, and/or 6 months. Full measures and methods are described in Supplementary Table S1, with supporting references listed in the Supplementary References.

### Statistical Analysis

Because this was a feasibility trial, formal power calculations were not required. The target sample size of 50 is adequate for estimating recruitment and dropout rates, calculating > 10 kg weight loss incidence, and assessing variance in weight loss and biomarkers for future sample size calculations for larger clinical effectiveness trials.^30^ Based on the commonly accepted approach that 15 to 30 subjects per group are required to derive estimates of centrality and spread statistics, a conservative sample of 50 participants was considered adequate to derive estimates of variance in the outcome measures likely to be used to calculate the sample size for a definitive trial, allowing for a 20% to 25% dropout. Analyses were performed using R software version 4.3.0 2023-04-21 (R Foundation for Statistical Computing, Vienna, Austria) and developed in RStudio version 2023.6.2.561 (Posit Software, PBC, Boston, MA). Categorical variables were described with frequencies and percentages, whereas continuous variables were described with median and interquartile range. Mann-Whitney U tests were used to compare median changes in secondary outcomes over time between groups. Fisher Exact test was used to examine differences in the frequency of counts across each group for categorical data. For the weight loss feasibility outcome, missing data were imputed by assuming that participants who dropped out or withdrew did not achieve the target weight loss. For secondary outcomes, no imputation was done for missing data. Statistical significance was set at *P* < 0.05.

## Results

### Participant Characteristics

Forty-nine participants (57% male, median age: 51 years, body mass index: 39 kg/m^2^, and eGFR: 57 ml/min per 1.73 m^2^) were recruited. Baseline anthropometric, clinical, biochemical, and functional measures are reported in Table 2. The leading cause of CKD was diabetic nephropathy. Multiple comorbidities and polypharmacy (regular use of 5 or more medications) were evident in both groups. Nine participants (4 in the LED group, and 5 in the UC group) were prescribed glucagon-like peptide-1 receptor agonists (GLP-1 RAs), contemporaneously (Supplementary Table S3). A detailed description of participant baseline demographics and medical history is provided in Supplementary Table S2.

**Table 2:** Changes in secondary outcome measures at 3- and 6-months in adults with obesity and chronic kidney disease from the SLOW-CKD feasibility study

Variables	Baseline		Median change 0–3 mos			Median change 0–6 mos		
	LED (*n* = 24)	UC (*n* = 25)	LED (*n* = 17)	UC (*n* = 20)	*P*-value	LED (*n* = 16)	UC (*n* = 22)	*P-*value
Anthropometric								
Weight (kg)	109 (103–124)	117 (102–132)	−10.0 (−13.3 to −7.3)	−0.5 (−2.3 to 1.8)	<0.001	−9.0 (−12 to −7)	0 (−4 to 2)	<0.001
Weight (%)	-	-	−9.3 (−11.4 to −7.0)	−0.5 (−2.0 to 1.2)	<0.001	−8.0 (−12 to −6)	0 (−3 to 2)	<0.001
Body mass index (kg/m^2^)	37 (35–44)	40 (36–43)	−3.1 (−4.8 to −2.9)	−0.17 (−0.7 to 0.6)	<0.001	−3.0 (−4.1 to −2.7)	0.1 (−1.2 to 0.7)	<0.001
Waist circumference (cm)	123 (114–136)	125 (117–138)	−5.9 (−8.5 to −1.7)	−1.7 (−4.8 to 1.7)	0.014	−10.0 (−12.9 to −6.0)	−2.5 (−5.1 to 1.8)	0.002
Clinical								
Systolic blood pressure (mm Hg)	131 (125–134)	128 (120–139)	−7 (−15 to −4)	−1 (−7 to 9)	0.018	−7 (−13 to 1)	−1 (−9 to 11)	0.3
Diastolic blood pressure (mm Hg)	81 (78–87)	80 (75–83)	−6 (−7 to 2)	1 (−2 to 4)	0.072	−3 (−5 to 0)	1 (−3 to 5)	0.1
Kidney measures								
Urinary PCR (mg/mmol)	101 (40–224)	76 (35–151)	-	-	-	−17 (−59 to 18)A	−13 (−37 to 64)A	0.3
Serum creatinine (g/mol)	111 (70–157)	112 (86–146)	-	-	-	−7 (−17 to −1)	3 (−5 to 14)	0.01
eGFR (ml/min per 1.73 m^2^)B								
CKD-EPI CR 2009	54 (37–95)^a^	55 (45–75)	-	-	-	4 (1–7)	−2 (−6 to 4)	0.06
CKD-EPI CR 2021	57 (40–98)^a^	59 (47–75)	-	-	-	4 (1–7)	−2 (−6 to 4)	0.06
CKD-EPI Cys-Cr	47 (29–75)^b^	41 (32–59)^c^	-	-	-	11 (0–35)^d^	7 (−2 to 20)^e^	0.2
Unadjusted eGFR (ml/min)B								
CKD-EPI CR 2009	64 (48–101)^f^	69 (56–83)^a^	-	-	-	4 (2–6)^g^	−2 (−7 to 5)^h^	0.2
CKD-EPI CR 2021	65 (49–95)^e^	72 (59–84)^i^	-	-	-	4 (2–6)^g^	−3 (−7 to 6)^h^	0.2
CKD-EPI Cys-Cr	60 (37–90)^h^	54 (41–63)^f^	-	-	-	14 (−1 to 44)^g^	8 (−1 to 25)^j^	0.4
Functional Measures								
6-min walk (m)	429 (345–474)	435 (335–515)^a^	63 (5–104)^j^	30 (−4 to 46)^f^	0.038	78 (64–173)^k^	10 (−71 to 47)^c^	0.001
30 s sit to stand	13.0 (9.3–14.8)^i^	10.5 (8.0–13.5)^l^	−0.50 (−1.0 to 1.75)^m^	1.0 (1.0–3.0)^d^	0.036	2.0 (1.0–4.0)^d^	1.0 (0.0–3.0)^c^	0.3
Hand grip strength (kg)	33 (29–41)	35 (24–43)^l^	1.3 (−1.5 to 6.7)^j^	1.3 (−2.1 to 3.5)^f^	0.500	4.2 (−2.8 to 6.0)^m^	2.8 (0.0–5.2)^i^	>0.9
Quality of life								
EQ-5D-5L (VAS)	50 (40–60)^c^	50 (29–65)	10 (0–18)^m^	10 (0–25)^e^	0.786	20 (5–33)^d^	15 (1–25)c	0.3
EQ-5D-5L (Index)	0.889 (0.766–0.945)^a^	0.880 (0.653–0.945)^i^	0.003 (−0.385 to 0.055)^e^	0.000 (−0.428 to 0.060)^h^	0.577	0.019 (0.000–0.061)^j^	−0.013 (−0.090 to 0.049)^i^	0.1

CKD-EPI, Chronic Kidney Disease Epidemiology Collaboration equation; CKD-EPI CR 2009, CKD-EPI creatinine equation 2009; CKD-EPI CR 2021, CKD-EPI creatinine equation 2021; CKD-EPI Cys-Cr, CKD-EPI creatinine-cystatin C equation 2021; eGFR, estimated glomerular filtration rate; EQ-5D-5L, European Quality of Life Five-Dimension Five Levels scale; LED, low energy diet; PCR, protein-creatinine ratio; UC, usual care; VAS, visual analogue scale.

^A^Expressed as percentage change.

^B^Excludes eGFR > 120 ml/min per 1.73 m^2^ at baseline.

Sample size: ^a^*n* = 23, ^b^*n* = 21, ^c^*n* =19, ^d^*n* = 13, ^e^*n* = 17, ^f^*n* = 18, ^g^*n* = 12, ^h^*n* = 20, ^i^*n* = 22, ^j^*n* = 16, ^k^*n* = 15, ^l^*n* = 24, and ^m^*n* = 14.

Data expressed as median and interquartile range.

### Primary Outcomes

**Figure 1:**
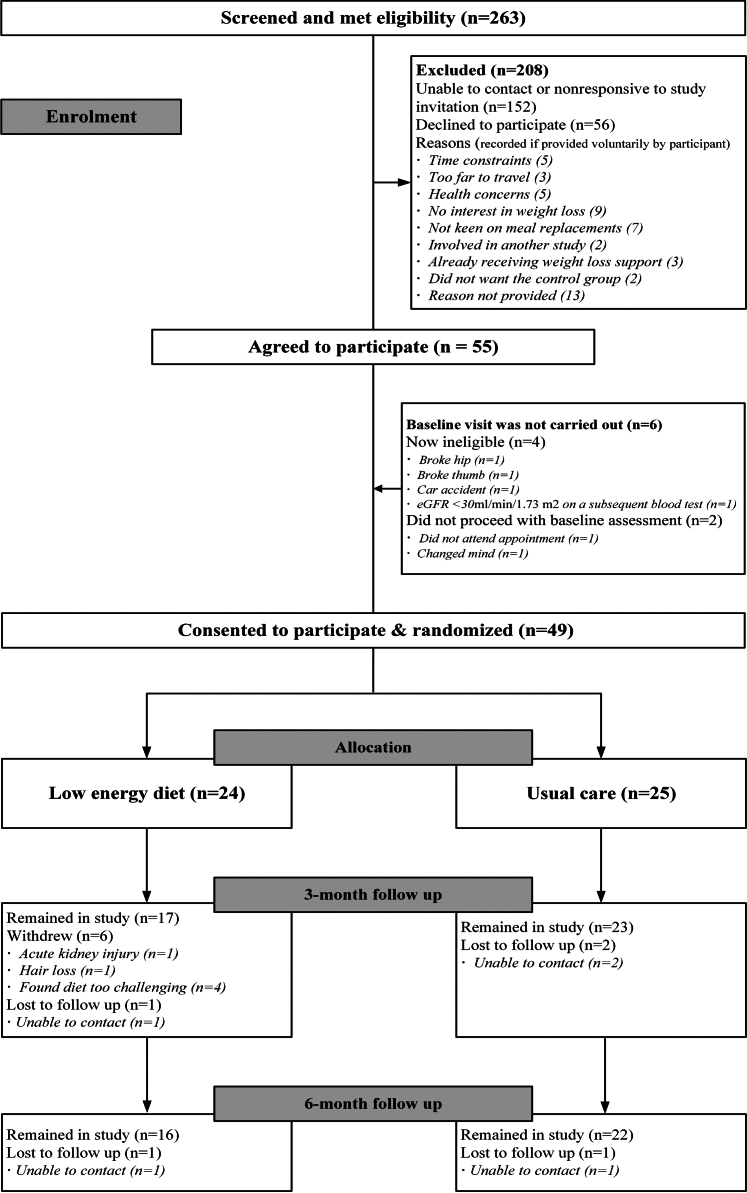
CONSORT diagram of the SLOW-CKD feasibility study in adults with obesity and chronic kidney disease.

In Table 1, we show that the SLOW-CKD feasibility study met the preset safety criteria and 2 of the 3 feasibility criteria, confirming the study as safe and feasible. Four possible study-related SAEs were recorded, with no significant difference between groups (Supplementary Table S4). The participant flow is described in Figure 1. Out of 107 eligible participants, 49 consented, resulting in a 46% recruitment rate. Thirty-eight participants (75%) completed the study; 88% of the UC group and 67% of the LED group (Table 1). Most LED group dropouts or withdrawals (88%) occurred before the 3-month visit. Across the study cohort, females had lower completion rate (37%) than males (63%).

### Secondary Outcomes

Hyperkalemia was the most common adverse event observed in both groups with similar frequencies (Supplementary Table S4). One participant in the LED group withdrew because of experiencing hair loss. Common side effects in the LED group included hunger, constipation, headache, light-headedness, and fatigue. These side effects were typically intermittent and confined to the initial weeks of the intervention.

**Figure 2:**
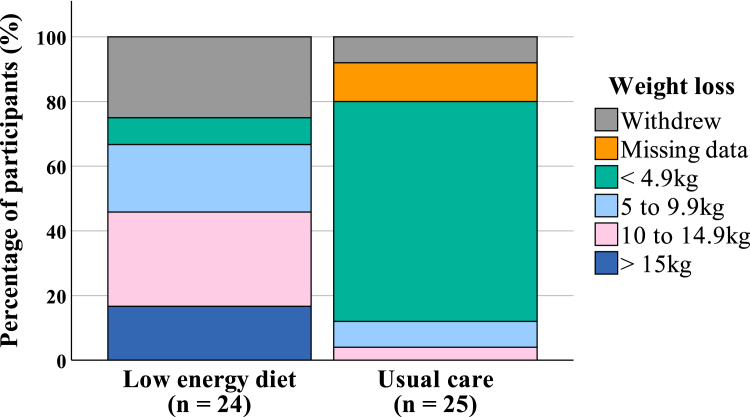
Absolute weight loss achieved by adults with obesity and chronic kidney from the SLOW-CKD feasibility study at 3 months by weight loss category.

Because of the feasibility trial design, the study was not powered to assess clinical effectiveness. Exploratory findings are presented in Table 2. Median weight change at 3 months was −10.0 kg in the LED group versus −0.5 kg in the UC group (*P* < 0.001). At 6 months, the median weight change was −9.0 kg in the LED group and 0 kg in the UC group (*P* < 0.001). At 3 months, a significantly higher proportion of participants in the LED group lost ≥10 kg compared with the UC group (*P* < 0.001) (Figure 2). The LED group had significantly greater improvements in waist circumference, systolic blood pressure, and 6-minute walk test time at 3 months; as well as waist circumference, 6-minute walk test distance, and creatinine at 6 months compared to UC. There was large variability across eGFR measures. Change in health-related quality-of-life measures did not differ between groups. Median visual analogue scale increased from baseline at 3 and 6 months in both groups; however, the index score remained unchanged, indicating minimal change in individual domain scores (Supplementary Table S5). Medication changes are detailed in Supplementary Table S6.

In Supplementary Table S7, we outline the data collected for completion rates, clinical parameters, biochemical markers, functional measures, and patient-reported outcomes. Completion rates were high (>90%) for most routine tests, anthropometric and clinical measures, as well as patient-administered surveys. Physical function measures were affected by injury concerns or contraindications to exercise, low baseline function, and participant reluctance. The ability to measure GFR was impacted by shortages of iohexol during the COVID-19 pandemic, resulting in only 8 participants being offered the measure, with just 1 electing to proceed.

## Discussion

In this feasibility study, we demonstrated that an LED combined with healthy eating and exercise support, under professional guidance, can be a safe and feasible weight loss approach for adults with stages G1 to G3b CKD. The intervention met safety criteria, with incidences of SAEs and adverse events being low and similar in both groups. During the LED phase, 2 participants were hospitalized for acute kidney injury and hypoglycemia, likely related to the study intervention. Hypoglycemia is a known risk of LEDs in people with diabetes.^31^ Despite regular support from health professionals, severe hypoglycemia occurred. This emphasizes the importance of monitoring by a professional trained in insulin adjustment when using LEDs and exercising caution in individuals with hypoglycemia unawareness.

Hyperkalemia was an adverse event experienced by participants, at similar rates in both groups. These findings contribute to the body of evidence that LEDs need to be used with caution in individuals with a history of hyperkalemia.^23^^,^^32^ Three participants in the LED group experienced a temporary decline in kidney function during the LED phase. Although the exact cause was not known, kidney function was restored after increasing fluid intake. Education on maintaining hydration and blood pressure monitoring may help manage this risk.

Study feasibility was established, by meeting 2 of the 3 feasibility criteria. Our recruitment rate target was exceeded, potentially indicating a strong interest in this weight management approach among those with CKD. This aligns with patient-reported priorities for CKD research, including diet and lifestyle interventions to prevent CKD progression,^33^ and weight loss being a common health goal of people living with CKD.^34^ Our study’s retention rate in the LED group fell short of our target, whereas retention in the UC group was high. Reasons for noncompletion of the LED phase of the study included difficulty to follow the diet, a dislike or intolerance of meal replacements, and hair loss. This suggests that, even with professional guidance and support, LEDs can be challenging for some people. Overall, our retention rates are comparable to those reported in other studies involving VLEDs or LEDs (48%–100%),^19^ self-management interventions for CKD (61%–89%),^35^ and GLP1-RA interventions. Continuation of GLP1-RA treatment has ranged from 75% to 100% in clinical trials involving adults with CKD^16^^,^^36^ and 35% to 63% in clinical practice in non-CKD populations.^37^ These findings highlight that not everyone will choose to begin or succeed with intensive weight loss diets or medications, supporting the need for flexible, stigma-free, supportive, patient-centered obesity management pathways in those with CKD. Our data will also help inform sample size calculations for future effectiveness trials. Further trials should account for at least a 30% dropout rate in the LED group.

Just under half of LED participants lost ≥ 10 kg at 3 months, meeting the weight loss feasibility target. Weight regains during the maintenance phase were minimal, with a median weight loss of 9 kg maintained at 6 months. The study used a flexible LED approach with meal replacements and low energy foods, rather than a mandatory total diet replacement. Our results are comparable to the studies targeting weight loss for diabetes remission using a total diet replacement LED program such as the DiRECT-UK^20^ (−10 kg at 12 months), DiRECT-Aus^38^ (−11% of body weight at 3 months, −8% body weight at 12 months), DIADEM-I^39^ (−12 kg at 12 months), and STANDby^40^ (−7 kg at 11–19 weeks) studies. We have demonstrated that a flexible LED with partial meal replacements can achieve weight loss similar to that of 825 to 950 kcal/d total diet replacement approaches. Our weight loss results, like those in the DiRECT studies, exceed those of an Australian study using a partial meal replacement LED, where participants lost an average of 5.7 kg at 3 months without access to a dietitian, exercise specialist, or behavioral therapy.^41^ This underscores the value of multidisciplinary input in supporting people’s LED weight loss journey.

The significant weight loss achieved by participants in the LED group was also accompanied by improvements in waist circumference, and blood pressure at 3 months compared with the UC. At 6 months, significant intervention effects were observed for waist circumference but not for systolic blood pressure, diastolic blood pressure, or urinary protein-to-creatinine ratio. There was large variability across eGFR measures. This supports the current literature that evaluating kidney function during weight loss is challenging because usual GFR markers, including creatinine and to a lesser extent cystatin C can be influenced by body weight and muscle mass.^42^ Our results are promising, suggesting that LEDs can target risk factors of cardiovascular disease and CKD progression (body weight, waist circumference, blood pressure). However, it was not clear from our study whether LEDs can improve markers of CKD progression through preservation of eGFR or reductions in proteinuria.

The LED program did not negatively impact quality-of-life in participants who remained in the study, because index scores were maintained, and visual analogue scale score improved in both groups. In contrast to the belief that severely restricted diets are unappealing and hard to follow,^43^ our findings agree with research confirming that adherence to, and tolerance of such diets are feasible.^44^^,^^45^ However, it remains unknown whether LED participants who did not complete the study dropped out or withdrew because of a negative impact on their quality of life. Functional measures and the 6-minute walk test remained stable at 3 months and improved at 6 months in the LED group, suggesting that LEDs did not negatively impact neuromuscular fitness and strength, or cardiovascular fitness. This is consistent with the literature that emphasizes the role of exercise and preference of LEDs over VLEDs for muscle preservation,^19^ and a lower risk of frailty.^46^

A strength of this study is its LED intervention design, which incorporated consumer input and offered a flexible LED prescription with both food options and meal replacements. This flexibility, noted in our earlier work^23^ and confirmed by similar studies,^40^ helps reduce hunger, minimize side effects, and support social interactions. The study is also strengthened by the pragmatic inclusion criteria, which included patients living with both type 1 and type 2 diabetes requiring insulin. It is the first RCT to specifically explore LED use in individuals with CKD.

The study has several limitations. The study was a single-blinded, which may have resulted in expectation bias in the LED group or a lack of accountability in the UC group. The small sample of Australian adults with stages G1 to G3b CKD may limit generalizability to other ethnicities and to individuals with advanced CKD, kidney transplant recipients, or those receiving dialysis. The study did not investigate body composition measures and changes in factors such as extracellular water, intracellular water (marker of muscle mass) and fat mass, each of which may influence adverse events (e.g., electrolytes) and quality of life measures. The study did not use an intention-to-treat analyses for the secondary analyses; therefore, results may be biased toward the intervention. In addition, the study was not powered for secondary outcomes and did not consider issues with multiple hypothese testing. The *P*-values have not been corrected or adjusted. *P*-values close to 0.05 have the potential of being false-positive results. The study follow up was 6 months; therefore, we cannot determine whether the weight loss would have continued or been maintained longer term. Longer-term studies involving all stages of CKD are needed.

The study did not exclude participants who were prescribed GLP-1 RAs, which may have influenced weight loss results. However, the study did exclude those who had recently lost ≥ 5% of their body weight, including those who had lost weight while taking these medications. Few participants were prescribed these medications, and mostly at lower doses for diabetes, and most-faced supply issues because of global shortages. The emergence of new GLP-1 RAs for the treatment of type 2 diabetes and obesity holds promise for those with CKD, and reduces the risk of kidney disease progression in patients with type 2 diabetes and CKD.^15^ It is important to emphasize that in clinical practice, these medications should not be prescribed in isolation but rather in conjunction with a balanced weight loss approach involving diet and exercise support. According to the UK National Health Service prescribing policy, these can only be prescribed in conjunction with a diet and physical activity weight management service.^47^

Encouragingly, our LED results have demonstrated a similar magnitude of weight loss achieved with semaglutide within the CKD population. A recent RCT found that, semaglutide 2.4 mg/wk in 101 adults with CKD without diabetes led to a mean weight loss 10.2 kg and a 48.6% reduction in urinary albumin-to-creatinine ratio over 24 weeks, compared with a 1.2 kg weight loss and 7.4% increase in urinary albumin-to-creatinine ratio in the placebo group.^36^ Future diet and/or exercise RCTs that do not include pharmacologic interventions should stratify groups at baseline based on the routine use of these medications to control for their effects. A recent study in adults with type 2 diabetes has demonstrated greater reductions in body weight and fat mass following a 12-week VLED of 800 kcal/d or VLED combined with semaglutide, compared with semaglutide alone.^48^ Studies have also shown that patients who followed a VLED were more likely to keep weight off with pharmacotherapy support,^49^ and when drug trials have used pharmacotherapy adjunct to lifestyle and behavioral support, they have achieved greater weight loss results.^14^ In future trials, it would be interesting to investigate the effects of use of LEDs in combination with GLP1-RAs on health outcomes and long-term weight maintenance in people with CKD and obesity. Studies should examine the individual and combined effects of these interventions on kidney outcomes, as well as physical function, diet quality, quality of life, patient experience, adherence and cost-effectiveness. The results from these trials will assist clinicians and policymakers to develop care pathways that can be personalized to individuals’ health and weight loss goals.

## Conclusion

Managing excess body weight in the CKD population is a health priority. An LED combined with healthy eating and exercise support under professional guidance has been demonstrated to be a safe and effective weight loss option for adults with CKD Kidney Disease: Improving Global Outcomes stages G1 to G3b. Our results have demonstrated feasibility for an adequately powered RCT to examine the effectiveness of an LED intervention on cardiometabolic risk and CKD progression.

## Disclosure

MMC and HLMac received Optifast products, donated by Nestlé Health Sciences Australia for a previous research study. DWJ is supported by an Australian National Health and Medical Research Council Leadership Investigator Grant (APP1194485). AKV receives grant support from a Queensland Advancing Clinical Research Fellowship and an NHMRC Emerging Leader Grant (APP1196033) and is an Associate Editor of Kidney International Reports. Travel/accommodation support was provided to KH from AstraZeneca in June 2022. All the other authors declared no competing interests.

## Supplementary Material

Supplementary File (PDF) Supplementary References. Table S1. Secondary outcome measures in adults with obesity and chronic kidney disease from the SLOW-CKD feasibility study measured at each study time point. Table S2. Participant baseline characteristics of adults with obesity and chronic kidney disease from the SLOW-CKD feasibility study. Table S3. Glucagon-like peptide-1 receptor agonist medication use and weight change in adults with obesity and chronic kidney disease from the SLOW-CKD feasibility study. Table S4. Adverse events recorded adults with obesity and chronic kidney disease from the SLOW-CKD feasibility study. Table S5. Quality of life dimensions in adults with obesity and chronic kidney disease from the SLOW-CKD feasibility, measured by the European Quality of Life Five-Dimension Five Levels scale (EQ-5D-5L). Table S6. Medication and dose changes in adults with obesity and chronic kidney disease from SLOW-CKD feasibility study. Table S7. Feasibility of measuring biochemical, clinical, dietary, and patient-centered outcomes in adults with obesity and chronic kidney disease from the SLOW-CKD feasibility study. CONSORT Checklist.

## References

[1] Xu H., Kuja-Halkola R., Chen X., Magnusson P.K.E., Svensson P., Carrero J.J. (2019). Higher body mass index is associated with incident diabetes and chronic kidney disease independent of genetic confounding. Kidney Int.

[2] Hsu C.Y., McCulloch C.E., Iribarren C., Darbinian J., Go A.S. (2006). Body mass index and risk for end-stage renal disease. Ann Intern Med.

[3] Dierkes J., Dahl H., Lervaag Welland N. (2018). High rates of central obesity and sarcopenia in CKD irrespective of renal replacement therapy - an observational cross-sectional study. BMC Nephrol.

[4] Evangelista L.S., Cho W.-K., Kim Y. (2018). Obesity and chronic kidney disease: a population-based study among South Koreans. PLoS One.

[5] Afshin A., Forouzanfar M.H., Reitsma M.B. (2017). Health effects of overweight and obesity in 195 countries over 25 years. N Engl J Med.

[6] Kidney Disease: Improving Global Outcomes (KDIGO) CKD Work Group (2024). KDIGO 2024 Clinical practice guideline for the evaluation and management of chronic kidney disease. Kidney Int.

[7] National Institute for Health and Care Excellence (Published 2021). Chronic kidney disease: assessment and management. https://www.nice.org.uk/guidance/ng203.

[8] Conley M.M., McFarlane C.M., Johnson D.W., Kelly J.T., Campbell K.L., MacLaughlin H.L. (2021). Interventions for weight loss in people with chronic kidney disease who are overweight or obese. Cochrane Database Syst Rev.

[9] Ikizler T.A., Burrowes J.D., Byham-Gray L.D. (2020). KDOQI clinical practice guideline for nutrition in CKD: 2020 update. Am J Kidney Dis.

[10] Colquitt J., Pickett K., Loveman E., Frampton G.K. (2014). Surgery for weight loss in adults. Cochrane Database Syst Rev.

[11] Cohen J.B., Lim M.A., Tewksbury C.M. (2019). Bariatric surgery before and after kidney transplantation: long-term weight loss and allograft outcomes. Surg Obes Relat Dis.

[12] Funes D.R., Montorfano L., Blanco D.G. (2022). Sleeve gastrectomy in patients with severe obesity and baseline chronic kidney disease improves kidney function independently of weight loss: a propensity score matched analysis. Surg Obes Relat Dis.

[13] Jastreboff A.M., Aronne L.J., Ahmad N.N. (2022). Tirzepatide once weekly for the treatment of obesity. N Engl J Med.

[14] Bergmann N.C., Davies M.J., Lingvay I., Knop F.K. (2023). Semaglutide for the treatment of overweight and obesity: a review. Diabetes Obes Metab.

[15] Colhoun H.M., Lingvay I., Brown P.M. (2024). Long-term kidney outcomes of semaglutide in obesity and cardiovascular disease in the SELECT trial. Nat Med.

[16] Perkovic V., Tuttle K.R., Rossing P. (2024). Effects of semaglutide on chronic kidney disease in patients with type 2 diabetes. N Engl J Med.

[17] Kim D.D., Hwang J.H., Fendrick A.M. (2024). Balancing innovation and affordability in anti-obesity medications: the role of an alternative weight-maintenance program. Health Aff Sch.

[18] Therapeutic Good Administration (Updated February 12, 2025). Australian Government Department of Health and Aged Care. About the Ozempic (semaglutide) shortage 2022-2024. https://www.tga.gov.au/safety/shortages/information-about-major-medicine-shortages/about-ozempic-semaglutide-shortage.

[19] Brown A., Leeds A.R. (2019). Very low energy and low-energy formula diets: effects on weight loss, obesity co-morbidities and type 2 diabetes remission-an update on the evidence for their use in clinical practice. Nutr Bull.

[20] Lean M.E., Leslie W.S., Barnes A.C. (2018). Primary care-led weight management for remission of type 2 diabetes (DiRECT): an open-label, cluster-randomised trial. Lancet.

[21] Sattar N., Taheri S., Astling D.P. (2023). Prediction of cardiometabolic health through changes in plasma proteins with intentional weight loss in the DiRECT and DIADEM-I randomized clinical trials of type 2 diabetes remission. Diabetes Care.

[22] Christensen P., Bliddal H., Riecke B.F., Leeds A.R., Astrup A., Christensen R. (2011). Comparison of a low-energy diet and a very low-energy diet in sedentary obese individuals: a pragmatic randomized controlled trial. Clin Obes.

[23] Conley M., Mayr H.L., Hoch M., Johnson D.W., Viecelli A.K., MacLaughlin H. (2024). Acceptability, adherence, safety and experiences of low energy diets in people with obesity and chronic kidney disease: a mixed methods study. J Ren Nutr.

[24] Levey A.S., Eckardt K.-U., Dorman N.M. (2020). Nomenclature for kidney function and disease: report of a Kidney Disease: improving Global Outcomes (KDIGO) Consensus Conference. Kidney Int.

[25] Harris P.A., Taylor R., Minor B.L. (2019). The REDCap consortium: building an international community of software platform partners. J Biomed Inform.

[26] Heart Foundation Dietary position statement, heart healthy eating patterns. https://assets.contentstack.io/v3/assets/blt8a393bb3b76c0ede/bltfdcaaee65e5c5af0/659e0be9a0eb30216a4ff37d/Nutrition_Position_Statement_-_HHEP_FINAL-3.pdf.

[27] Australian Government, National Health and Medical Research Council, Department of Health and Aged Care Australian dietary guidelines. Advice about the amount and kinds of foods that we need to eat for health and wellbeing. https://www.eatforhealth.gov.au.

[28] Australian Government, Department of Health and Aged Care Physical activity and exercise guidelines for all Australians. https://www.health.gov.au/topics/physical-activity-and-exercise/physical-activity-and-exercise-guidelines-for-all-australians.

[29] Koufaki P., Greenwood S., Painter P., Mercer T. (2015). The BASES expert statement on exercise therapy for people with chronic kidney disease. J Sports Sci.

[30] Cocks K., Torgerson D.J. (2013). Sample size calculations for pilot randomized trials: a confidence interval approach. J Clin Epidemiol.

[31] Brown A., Dornhorst A., McGowan B. (2020). Low-energy total diet replacement intervention in patients with type 2 diabetes mellitus and obesity treated with insulin: a randomized trial. BMJ Open Diabetes Res Care.

[32] Woods J.E., Snelson A., Kok J. (2024). Safety and efficacy of very low calorie diet in patients receiving haemodialysis therapy. Clin Kidney J.

[33] Hemmelgarn B.R., Pannu N., Ahmed S.B. (2017). Determining the research priorities for patients with chronic kidney disease not on dialysis. Nephrol Dial Transplant.

[34] Jegatheesan D.K., Perez W.F.P., Brown R. (2023). Goal attainment scale as an outcome measure in a randomized controlled trial of lifestyle interventions: FR-PO818. J Am Soc Nephrol.

[35] Welch J.L., Johnson M., Zimmerman L., Russell C.L., Perkins S.M., Decker B.S. (2015). Self-management interventions in Stages 1 to 4 chronic kidney disease: an integrative review. West J Nurs Res.

[36] Apperloo E.M., Gorriz J.L., Soler M.J. (2025). Semaglutide in patients with overweight or obesity and chronic kidney disease without diabetes: a randomized double-blind placebo-controlled clinical trial. Nat Med.

[37] Rodriguez P.J., Zhang V., Gratzl S. (2025). Discontinuation and reinitiation of dual-labeled GLP-1 receptor agonists among US adults with overweight or obesity. JAMA Netw Open.

[38] Hocking S.L., Markovic T.P., Lee C.M.Y., Picone T.J., Gudorf K.E., Colagiuri S. (2024). Intensive lifestyle intervention for remission of early type 2 diabetes in primary care in Australia: DiRECT-Aus. Diabetes Care.

[39] Taheri S., Zaghloul H., Chagoury O. (2020). Effect of intensive lifestyle intervention on bodyweight and glycaemia in early type 2 diabetes (DIADEM-I): an open-label, parallel-group, randomised controlled trial. Lancet Diabetes Endocrinol.

[40] Sattar N., Welsh P., Leslie W.S. (2023). Dietary weight-management for type 2 diabetes remissions in South Asians: the South Asian diabetes remission randomised trial for proof-of-concept and feasibility (STANDby). Lancet Reg Health S East Asia.

[41] Khoo C.L., Chimoriya R., Simmons D., Piya M.K. (2023). Partial meal replacement for people with type 2 diabetes: 2-year outcomes from an Australian general practice. Aust J Prim Health.

[42] von Scholten B.J., Persson F., Svane M.S., Hansen T.W., Madsbad S., Rossing P. (2017). Effect of large weight reductions on measured and estimated kidney function. BMC Nephrol.

[43] Maston G., Franklin J., Gibson A.A. (2020). Attitudes and approaches to use of meal replacement products among healthcare professionals in management of excess weight. Behav Sci (Basel).

[44] Campbell K., Peddie M., Ashton N. (2024). Experiences and acceptability of a weight loss intervention for diabetes (diabetes remission clinical trial-DiRECT) in Aotearoa New Zealand: a qualitative study within a pilot randomised controlled trial. Nutrients.

[45] Rehackova L., Rodrigues A.M., Thom G. (2022). Participant experiences in the Diabetes REmission Clinical Trial (DiRECT). Diabet Med.

[46] Johansen K.L., Dalrymple L.S., Delgado C. (2014). Association between body composition and frailty among prevalent hemodialysis patients: a US Renal Data System special study. J Am Soc Nephrol.

[47] National Health Service, National Institute for Health and Care Excellence Appraisal consultation document semaglutide for managing overweight and obesity. https://www.nice.org.uk/guidance/ta875/documents/129#:%7E:text=2.1%20Semaglutide%20(Wegovy%2C%20Novo%20Nordisk,%2Fkg2%20(overweight)%20in%20the.

[48] Anyiam O., Phillips B., Quinn K. (2024). Metabolic effects of very-low calorie diet, semaglutide, or combination of the two, in individuals with type 2 diabetes mellitus. Clin Nutr.

[49] Sumithran P., Prendergast L.A., Haywood C.J., Houlihan C.A., Proietto J. (2016). Review of 3-year outcomes of a very-low-energy diet-based outpatient obesity treatment programme. Clin Obes.

