# Supplementary material for: Low Energy Diets for Obesity and CKD (SLOW-CKD Randomized Feasibility Study)
Source: Kidney Int Rep. 2025 Apr 21;10(7):2153–64. doi: 10.1016/j.ekir.2025.04.021 (PMC12266257; doi:10.1016/j.ekir.2025.04.021)
Supplement: Supplementary File (PDF) — Supplementary References. Table S1. Secondary outcome measures in adults with obesity and chronic kidney disease from the SLOW-CKD feasibility study measured at each study time point. Table S2. Participant baseline characteristics of adults with obesity and chronic kidney disease from the SLOW-CKD feasibility study. Table S3. Glucagon-like peptide-1 receptor agonist medication use and weight change in adults with obesity and chronic kidney disease from the SLOW-CKD feasibility study. Table S4. Adverse events recorded adults with obesity and chronic kidney disease from the SLOW-CKD feasibility study. Table S5. Quality of life dimensions in adults with obesity and chronic kidney disease from the SLOW-CKD feasibility, measured by the European Quality of Life Five-Dimension Five Levels scale (EQ-5D-5L). Table S6. Medication and dose changes in adults with obesity and chronic kidney disease from SLOW-CKD feasibility study. Table S7. Feasibility of measuring biochemical, clinical, dietary, and patient-centered outcomes in adults with obesity and chronic kidney disease from the SLOW-CKD feasibility study. CONSORT Checklist. [file mmc1.pdf]

Supplementary Material

Table S1. Secondary outcome measures in adults with obesity and chronic kidney disease from the of the SLOW-CKD feasibility study measured at each study timepoint.

Table S2. Participant baseline characteristics of adults with obesity and chronic kidney disease from the SLOW-CKD feasibility study.

Table S3. Glucagon-like peptide-1 receptor agonist medication use and weight change in adults with obesity and chronic kidney disease from the SLOW-CKD feasibility study.

Table S4. Adverse events recorded adults with obesity and chronic kidney disease from the SLOW-CKD feasibility study.

Table S5. Quality of life dimensions in adults with obesity and chronic kidney disease from the SLOW-CKD feasibility, measured by the European Quality of Life Five-Dimension Five Levels scale (EQ-5D-5L).

Table S6. Medication and dose changes in adults with obesity and chronic kidney disease from SLOW-CKD feasibility study.

Table S7. Feasibility of measuring biochemical, clinical, dietary, and patient-centered outcomes in adults with obesity and chronic kidney disease from the SLOW-CKD feasibility study.

CONSORT Checklist

Table S1. Secondary outcome measures in adults with obesity and chronic kidney disease from the of the SLOW-CKD feasibility study measured at each study timepoint.

**Outcome**	**Timepoints (month)**	**Description**
Clinical measures	0, 3, 6	Systolic and diastolic blood pressure were measured three times while the participant was seated in a chair after a 10-minute rest, with two minutes between each measurement. The mean of the two closest measures was recorded.
Anthropometric	0, 3, 6	Measurements were taken with shoes off and in light clothing. Height was measured to the nearest centimetre using a stadiometer, and body weight was measured to the nearest 0.1 kg using calibrated electronic scales. The average of three waist circumference measurements using standardised measures to the nearest 0.5 centimetre was recorded.[S1] Body mass index was calculated to the nearest 0.1 $kg/m.^2$
Laboratory measures	0, 6	Blood and urine samples were obtained using standardized protocols. Analyses for serum creatinine, potassium, blood lipids, and urinary markers were undertaken on the same day using standard laboratory methods.
Glomerular filtration rate (GFR)	0	Measured GFR was examined using iohexol clearance, following Queensland Health standard protocols. To test feasibility of collecting this measure a target of 16 measured GFRs was set. Estimated GFR was examined using the CKD Epidemiology Collaboration Creatinine 2009 [S2] and 2021 equations, as well as the CKD Epidemiology Collaboration Creatinine-Cystatin C 2021 equation.[S3] These were adjusted for body surface area and calculated without indexing, using actual body surface area.
Health-related quality of life	0, 3, 6	Participants completed the European Quality of Life Five-Dimension Five Levels scale (EQ-5D-5L). Scores were generated using validated Australian data sets.[S4]
Functional capacity	0, 3, 6	The 6-minute walk test was conducted using a standardized protocol on a 20 to 30m track to assess exercise capacity. [S1] The result of the test was recorded as the distance walked, rounded to the nearest meter.
Muscular strength and endurance	0,3,6	The 30 second sit-to-stand test (number) was used to assess functional lower limb muscular endurance using a standardized protocol. [S1] Hand grip strength was assessed using a grip dynamometer, three times in both hands with the highest result in kg for the dominant hand. [S1]
Medication	0, 3, 6	All types and frequencies of medications were collected from medical records and confirmed via self-report and any changes to medications recorded.
Other measures		Dietary intake, sleep, fatigue, and participant experiences were also measured but not reported here. Surveys assessing quality of life, sleep, and fatigue were self-administered and sent via email using REDCap software the day before participants' appointments for online completion. If participants did not complete the surveys online, they were given a paper version to fill out at their appointment. Dietary surveys were completed by participants with researcher support at participants appointments.

Table S2. Participant baseline characteristics of adults with obesity and chronic kidney disease from the SLOW-CKD feasibility study.

Characteristic	Overall (n=49)	LED (n=24)	UC (n=25)
Age, years median	51 (43, 63)	51 (41, 62)	54 (49, 63)
Gender (Male)	28 (57)	14 (58)	14 (56)
Smoking status			
Current	5 (10)	3 (13)	2 (8.0)
Former	19 (39)	9 (38)	10 (40)
Never	25 (51)	12 (50)	13 (52)
**Primary cause of kidney disease**			
Diabetic nephropathy	16 (33)	7 (29)	9 (36)
Hypertension / vascular	4 (8.2)	1 (4.2)	3 (12)
Glomerulonephritis	8 (16)	3 (13)	5 (20)
Reflux nephropathy	15 (31)	12 (50)	3 (12)
Polycystic kidney disease	3 (6.1)	1 (4.2)	2 (8.0)
Other	1 (2.0)	0 (0)	1 (4.0)
Unknown	2 (4.1)	0 (0)	2 (8.0)
**Co-morbidities**			
Cardiovascular disease	15 (31)	7 (29)	8 (32)
Cerebrovascular disease	4 (8.2)	1 (4.2)	3 (12)
Dyslipidaemia	34 (69)	14 (58)	20 (80)
Hypertension	42 (86)	23 (96)	19 (76)
Respiratory disease	19 (39)	8 (33)	11 (44)
Peripheral vascular disease	8 (16)	3 (13)	5 (20)
**Diabetes (total)**	27 (55)	11 (46)	16 (64)
Type 1	3 (12)	0 (0)	3 (19)
Type 2	23 (88)	10 (100)	13 (81)
Type 2 requiring insulin	14 (29)	6 (25)	8 (32)
**Medication use**			
Insulin	14 (29)	6 (25)	8 (32)
Oral Antihyperglycemic agent	22 (45)	12 (50)	10 (40)
Injectable antihyperglycemic agent (GLP-1 RA)	7 (14)	3 (13)	4 (16)
Antihypertensive	44 (90)	22 (92)	22 (88)
Diuretic	7 (14)	4 (17)	3 (12)
Lipid lowering	30 (61)	14 (58)	16 (64)
Total number	7 (5, 12)	6 (4, 9)	6 (4.0, 10)
**Biochemistry**			
Sodium, mmol/L	140 (138, 142)	140 (138, 140)	140 (138, 142)
Potassium, mmol/L	4.4 (4.1, 4.8)	4.55 (4.2, 4.9)	4.3 (4.1, 4.8)
Phosphate, mmol/L	3.53 (3.03, 3.84)	3.53 (3.32, 4.15)	3.5 (2.88, 3.75)

Data are Median, (interquartile range) or number (%). Data were collected from patients' electronic medical records and confirmed through self-report by participants. Abbreviations: LED, low energy diet; GLP-1 RA, glucagon-like peptide-1 receptor agonists; UC, usual care. Categorical variables are presented as count (percentage); percentages may not total 100 because of rounding.

Table S3. Glucagon-like peptide-1 receptor agonist medication use and weight change in adults with obesity and chronic kidney disease from the SLOW-CKD feasibility study.

ID	Medication	Indication	Dose	Group	Weight change 0-3m (kg)	Weight change 0-6m (kg)	Status
7	Dulaglutide	Diabetes	1.5 mg weekly injection	LED	N/A	N/A	Prescribed prior to study. Withdrew week 2 from study.
44	Liraglutide	Obesity	0.6 / 2.4 mg daily injection	LED	-2.5	N/A	Private script for weight loss, started 2 weeks prior to study. Started at 0.6 mg, titrated up to 2.4 mg. Ceased week 2 of study due to side effects. Withdrew week 24 from study.
26	Semaglutide	Diabetes	0.5 mg weekly injection	LED	N/A	N/A	Prescribed prior to study. Withdrew week 6 from study.
52	Semaglutide	Diabetes	0.5 mg weekly injection	LED	-20.1	-26.6	Prescribed prior to study. Supply issues delayed use, commenced week 6 of study. Started at 0.25 mg and titrated up to 0.5 mg.
29	Dulaglutide	Diabetes	1.5 mg weekly injection	UC	+0.8	+1.6	Prescribed at week 14 of study.
17	Semaglutide	Diabetes	0.5 mg weekly injection	UC	-10.0	-3.0	Prescribed prior to study. No supply at start of study, recommenced medication week 8 of study.
21	Semaglutide	Diabetes	0.25 / 0.5 mg weekly injection	UC	+8.2	+10.4	Prescribed prior to study started at 0.25 mg, increased to 0.5 mg - Side effects with 0.5 mg, dropped back to 0.25 mg.
54	Semaglutide	Diabetes	1.0 mg weekly injection	UC	+6.6	+7.7	Prescribed prior to study, started at 0.25 mg, titrated up to 1.0 mg.
39	Semaglutide	Diabetes	0.5 mg weekly	UC	+1.8	-25.0	Prescribed week 16 of the study, commenced LED program in the community week 12 of study.

LED, low energy diet; UC, usual care; N/A, not applicable.

Table S4. Adverse events recorded adults with obesity and chronic kidney disease from the SLOW-CKD feasibility study.

Characteristic	*Baseline to 3 months*			*3 months to 6 months*		
	**LED (n = 24)**	**UC (n = 25)**	***p* value**	**LED (n = 16)**	**UC (n = 22)**	***p* value**
**Serious adverse event**^						
Hospitalisations						
Hypoglycemia	1	1		0	1	
Acute kidney injury	1	0		0	0	
Total	2	1	0.6	0	1	>0.9
**Adverse event**#						
Decline in kidney function$	3	0		0	2	
Mild hyperkalemia&	1	1		3	3	
Moderate hyperkalemia+	0	0		0	1	
Hair loss	1	0		0	0	
Musculoskeletal injury	1	1		4	3	
Gastrointestinal upset^^	3	2		2	1	
Fall	1	1		0	0	
Total	11	4		9	9	

Abbreviations: LED, low energy diet; UC, usual care.
^defined as any possible study related adverse event that led to death, life-threatening adverse event, inpatient hospitalisation or prolongation of existing hospitalisation, persistent/ significant incapacity/substantial disruption of the ability to conduct normal life functions. #defined as any possible study related untoward medical occurrence, unintended disease or injury, or untoward clinical signs (including abnormal laboratory findings). $Defined as a decrease in eGFR and or increase in creatinine >10% from baseline but not classified as acute kidney injury. &Defined by a serum potassium level of 5.5 to 6.5 mmol/L. +Defined by a serum potassium level of > 6.5 mmol/L. ^^New onset of persistent (>1 week) of gastrointestinal upset including diarrhoea, constipation, abdominal pain or indigestion.

Table S5. Quality of life dimensions in adults with obesity and chronic kidney disease from the SLOW-CKD feasibility, measured by the European Quality of Life Five-Dimension Five Levels scale (EQ-5D-5L).

Dimension	Baseline LED[1] (n = 24)	Baseline UC[2] (n = 25)	Baseline Overall (n=49)	3 months LED (n= 17)	3 months UC (n = 20)	3 months Overall (n = 37)	6 months LED (n = 16)	6 months UC (n = 22)	6 months Overall (n = 38)
Mobility									
No problems	7 (30)	13 (52)	20 (42)	6 (35)	6 (30)	12 (32)	6 (37)	6 (27)	12 (31)
Slight problems	6 (26)	5 (20)	11 (23)	8 (47)	7 (35)	15 (40)	6 (37)	7 (32)	13 (34)
Moderate problems	6 (26)	4 (16)	10 (21)	3 (18)	5 (25)	8 (22)	4 (25)	8 (36)	12 (32)
Severe problems	4 (17)	3 (12)	7 (15)	0	1 (5)	1 (3)	0	1 (5)	1 (3)
Unable to walk about	0	0	0	0	1 (5)	1 (3)	0	0	0
Self-care									
No problems	17 (74)	20 (80)	37 (77)	14 (82)	17 (85)	31 (84)	14 (88)	17 (77)	31 (82)
Slight problems	6 (26)	4 (16)	10 (21)	3 (18)	2 (10)	5 (13)	2 (12)	4 (16)	6 (16)
Moderate problems	0	1 (4)	1 (2)	0	1 (5)	1 (3)	0	1 (4)	1 (3)
Severe problems	0	0	0	0	0	0	0	0	0
Unable to wash or dress	0	0	0	0	0	0	0	0	0
Usual activities									
No problems	7 (31)	12 (48)	19 (40)	8 (47)	8 (40)	16 (43)	10 (63)	11 (50)	21 (55)
Slight problems	9 (39)	8 (32)	17 (35)	8 (47)	7 (35)	15 (40)	5 (31)	5 (23)	10 (26)
Moderate problems	6 (26)	2 (8)	8 (17)	1 (6)	3 (15)	4 (11)	1 (6)	4 (18)	5 (13)
Severe problems	1 (4)	2 (8)	3 (6)	0	1 (5)	1 (3)	0	1 (5)	1 (3)
Unable to do usual activities	0	1 (4)	1 (2)	0	1 (5)	1 (3)	0	1 (5)	1 (3)
Pain / discomfort									
No pain/discomfort	6 (26)	4 (16)	10 (21)	3 (17)	4 (20)	7 (19)	4 (25)	2 (9)	6 (16)
Slight pain/discomfort	10 (43)	10 (40)	20 (42)	7 (41)	9 (45)	16 (43)	7 (44)	10 (45)	17 (45)
Moderate pain/discomfort	5 (22)	7 (28)	12 (25)	6 (35)	5 (25)	11 (30)	4 (25)	5 (23)	9 (24)
Severe pain/discomfort	2 (9)	3 (12)	5 (10)	1 (6)	2 (10)	3 (8)	1 (6)	5 (23)	5 (13)

Extreme pain/discomfort	0	1 (4)	1 (2)	0	0	0	0	0	1 (3)
Anxiety/depression									
Not anxious/depressed	12 (52)	11 (44)	23 (48)	9 (53)	7 (35)	16 (43)	12 (75)	12 (54)	24 (63)
Slightly anxious/depressed	6 (26)	4 (16)	10 (21)	4 (23)	5 (25)	9 (24)	3 (20)	3 (14)	6 (16)
Moderately anxious/depressed	4 (17)	7 (28)	11 (23)	3 (17)	6 (30)	9 (24)	1 (6)	4 (18)	5 (13)
Severely anxious/depressed	1 (4)	2 (8)	3 (6)	1 (6)	2 (10)	3 (8)	0	2 (9)	2 (5)
Extremely anxious/depressed	0	1 (4)	1 (2)	0	0	0	0	1 (5)	1 (3)

[1]LED, low energy diet; [2]UC, usual care;

Table S6. Medication and dose changes in adults with obesity and chronic kidney disease from SLOW-CKD feasibility study.

	Baseline		3 months		6 months	
**Number of prescribed medications**	**LED[1] (n = 24)**	**UC[2] (n = 25)**	**LED (n= 17)**	**UC (n = 23)**	**LED (n = 16)**	**UC (n = 22)**
Insulin (n, %)						
0	18 (75)	18 (72)	13 (76)	16 (70)	12 (75)	15 (68)
1	4 (17)	4 (16)	2 (12)	4 (17)	2 (12.5)	5 (23)
≥ 2	2 (8)	3 (12)	2 (12)	3 (13)	2 (12.5)	2 (9)
Dose reduction	-	-	4 (22)	1 (4)	2 (12)	0
Dose increase	-	-	0	1 (4)	1 (6)	2 (9)
OHA[3] (n, %)						
0	11 (46)	14 (56)	7 (41)	11 (48)	6 (37.5)	10 (46)
1	6 (25)	3 (12)	5 (29.5)	4 (17)	6 (37.5)	4 (18)
≥ 2	7 (29)	8 (32)	5 (29.5)	8 (35)	4 (25)	8 (36)
Dose reduction	-	-	0	0	0	0
Dose increase	-	-	0	0	0	1 (4)
Non-insulin - Injectable antihyperglycemic (n, %)						
0	20 (83)	21 (84)	16 (94)	18 (78)	13 (81)	17 (77.3)
1	4 (17)	4 (16)	1 (6)	5 (22)	3 (12.5)	5 (22.7)
≥ 2	0	0	0	0	0	0
Dose reduction	-	-	0	0	0	0
Dose increase	-	-	1 (6)	1 (4)	0	0
Antihypertensive (n, %)						
0	1 (4)	1 (4)	1 (6)	3 (13)	1 (6)	2 (9)
1	8 (33)	7 (28)	6 (35)	5 (22)	5 (31)	6 (27)
≥ 2	15 (63)	17 (68)	10 (59)	15 (65)	10 (63)	14 (64)
Dose reduction	-	-	2 (11)	3 (13)	3 (17)	0
Dose increase	-	-	1 (6)	1 (4)	0	2 (9)
Diuretic (n, %)						
0	19 (79)	23 (92)	15 (88)	21 (91)	14 (87.5)	19 (86)
1	5 (21)	2 (8)	2 (12)	2 (9)	2 (12.5)	3 (14)
≥ 2	0	0	0	0	0	0
Dose reduction	-	-	0	0	1 (6)	0
Dose increase	-	-	0	0	0	0
Lipid lowering (n, %)						
0	8 (33)	10 (40)	6 (35)	9 (39)	5 (31)	8 (36)
1	12 (50)	10 (40)	8 (47)	9 (39)	8 (50)	9 (41)
≥ 2	4 (17)	5 (20)	3 (18)	5 (21)	3 (19)	5 (23)
Dose reduction	-	-	0	0	1 (6)	0
Dose increase	-	-	0	0	0	0

Data is expressed as the number and percentage of participants prescribed each type of medication.
[1]LED, low energy diet;[2]UC, usual care; [3]OHA; Oral antihyperglycemic agent.

Table S7. Feasibility of measuring biochemical, clinical, dietary, and patient-centered outcomes in adults with obesity and chronic kidney disease from the SLOW-CKD feasibility study.

Outcome measure	Completion rate n (%)	Reasons for non-completion	Feedback from participants
3-month study visit	37/40 (93)	Two participants were unable to attend the 3-month appointment due to COVID-19 but wished to remain in the study. Another participant was unable to attend the 3-month appointment due to travel constraints but wished to remain in the study.	Free parking and flexible study appointments were strong enablers for attendance.
Measured glomerular filtration rate	1/8 (12.5%)	The supply of iohexol was impacted by the COVID-19 pandemic. The study was unable to source a supply until March 2023, so only eight people were offered participation rather than the intended target 16.	Participants did not wish to come in for a four-hour invasive procedure on a separate occasion from their baseline visit.
Blood measures		One person had a needle phobia and did not wish to have their blood taken.	Having blood collection at same place as appointment on the same day was an enabler.
Baseline	48/49 (98)		
3-month	36/37 (98)		
6-month	38/38 (100)		
Urinary measures			Having urine collection at same place as appointment on the same day was an enabler.
Baseline	49/49 (100)		
3-month	37/37 (100)		
6-month	38/38 (100)		
Blood pressure			
Baseline	49/49 (100)		
3-month	37/37 (100)		
6-month	38/38 (100)		
Height, weight, and waist circumference			All participants were happy to have their weight and waist circumference recorded and monitored.
Baseline	49/49 (100)		
3-month	37/37 (100)		
6-month	38/38 (100)		
European Prospective Investigation into Cancer and Nutrition (EPIC) – Food frequency questionnaire.		Four participants ran out of time and were unable to complete it during their appointment time.	Some found the questionnaire long and felt it might be subject to recall bias. Many needed clinician assistance to fill it out correctly and ensure completeness.
Baseline	47/49 (96)		
6-month	36/38 (95)		
Pittsburgh Sleep Quality Index (PSQI)		One participant ran out of time and was unable to complete it.	
Baseline	48/49 (98)		

3-month	37/37 (100)		
6-month	38/38 (100)		
Functional Assessment of Chronic Illness Therapy Scale (FACIT)		Two participants ran out of time and were unable to complete it at their baseline assessment and did not complete the electronic copy emailed to them.	
Baseline	47/49 (98)		
3-month	37/37 (100)		
6-month	38/38 (100)		
European Quality of Life Five-Dimension Five Levels scale		One participant out of time and was not unable to complete it at their baseline assessment and did not complete the electronic copy emailed to them.	Some issues completing surveys online – with requests often going to junk/spam mail folders.
Baseline	48/49 (98)		
3-month	37/37 (100)		
6-month	38/38 (100)		
Hand grip strength (dominant hand)		One participant at baseline, two at 3 months and three at 6 months did not complete the measure due to concerns related to hand, wrist, or shoulder injuries.	
Baseline	48/49 (98)		
3-month	35/37 (95)		
6-month	35/38 (92)		
30 Second sit to stand test		Three participants at baseline, and two participants at 3 and 6 months, were unable to stand safely without assistance and, therefore, could not perform the test.	
Baseline	46/49 (94)		
3-month	35/37 (95)		
6-month	36/38 (95)		
6-minute walk test		Two participants at baseline, 3 and 6 months were medically or physically unable or unsafe to perform the test. Two participants at 6 months expressed the six-minute walk test was challenging and to chose not to complete it.	
Baseline	47/49 (96)		
3-month	35/37 (95)		
6-month	34/38 (89)		

Supplementary References

S1. Coombes J ST. ESSA's Student Manual for Health, Exercise and Sport Assessment. Elsevier Health Sciences APAC; 2014.

S2. Levey AS, Stevens LA, Schmid CH, et al. A new equation to estimate glomerular filtration rate. Ann Intern Med. 2009;150:604-612. https://doi:10.7326/0003-4819-150-9-200905050-00006

S3. Inker LA, Eneanya ND, Coresh J, et al. New Creatinine- and Cystatin C-Based Equations to Estimate GFR without Race. N Engl J Med. Nov 4 2021;385:1737-1749. https://doi:10.1056/NEJMoa2102953

S4. Norman R, Mulhern B, Lancsar E, et al. The Use of a Discrete Choice Experiment Including Both Duration and Dead for the Development of an EQ-5D-5L Value Set for Australia. Pharmacoeconomics. 2023;41:427-438. https://doi:10.1007/s40273-023-01243-0

CONSORT 2010 checklist of information to include when reporting a pilot or feasibility trial*

Section/Topic	Item No	Checklist item	Reported on page No
**Title and abstract**			
	1a	Identification as a pilot or feasibility randomised trial in the title	1
	1b	Structured summary of pilot trial design, methods, results, and conclusions (for specific guidance see CONSORT abstract extension for pilot trials)	2
**Introduction**			
Background and objectives	2a	Scientific background and explanation of rationale for future definitive trial, and reasons for randomised pilot trial	3
	2b	Specific objectives or research questions for pilot trial	4
**Methods**			
Trial design	3a	Description of pilot trial design (such as parallel, factorial) including allocation ratio	5
	3b	Important changes to methods after pilot trial commencement (such as eligibility criteria), with reasons	n/a
Participants	4a	Eligibility criteria for participants	4,5
	4b	Settings and locations where the data were collected	4,5
	4c	How participants were identified and consented	5
Interventions	5	The interventions for each group with sufficient details to allow replication, including how and when they were actually administered	6,7,8,9
Outcomes	6a	Completely defined prespecified assessments or measurements to address each pilot trial objective specified in 2b, including how and when they were assessed	9, Table 1
	6b	Any changes to pilot trial assessments or measurements after the pilot trial commenced, with reasons	12, supplementary material
	6c	If applicable, prespecified criteria used to judge whether, or how, to proceed with future definitive trial	9, Table 1
Sample size	7a	Rationale for numbers in the pilot trial	9,10
	7b	When applicable, explanation of any interim analyses and stopping guidelines	n/a
Randomisation:			
Sequence generation	8a	Method used to generate the random allocation sequence	5
	8b	Type of randomisation(s); details of any restriction (such as blocking and block size)	5
Allocation concealment mechanism	9	Mechanism used to implement the random allocation sequence (such as sequentially numbered containers), describing any steps taken to conceal the sequence until interventions were assigned	5
Implementation	10	Who generated the random allocation sequence, who enrolled participants, and who assigned participants to interventions	5

Blinding	11a	If done, who was blinded after assignment to interventions (for example, participants, care providers, those assessing outcomes) and how	5
	11b	If relevant, description of the similarity of interventions	n/a
Statistical methods	12	Methods used to address each pilot trial objective whether qualitative or quantitative	9, Supp Table 1
**Results**			
Participant flow (a diagram is strongly recommended)	13a	For each group, the numbers of participants who were approached and/or assessed for eligibility, randomly assigned, received intended treatment, and were assessed for each objective	Figure 1
	13b	For each group, losses and exclusions after randomisation, together with reasons	Figure 1
Recruitment	14a	Dates defining the periods of recruitment and follow-up	Figure 1
	14b	Why the pilot trial ended or was stopped	n/a
Baseline data	15	A table showing baseline demographic and clinical characteristics for each group	Supp Table 2
Numbers analysed	16	For each objective, number of participants (denominator) included in each analysis. If relevant, these numbers should be by randomised group	Table 2
Outcomes and estimation	17	For each objective, results including expressions of uncertainty (such as 95% confidence interval) for any estimates. If relevant, these results should be by randomised group	n/a
Ancillary analyses	18	Results of any other analyses performed that could be used to inform the future definitive trial	Supp Table 7
Harms	19	All important harms or unintended effects in each group (for specific guidance see CONSORT for harms)	Supp Table 4
	19a	If relevant, other important unintended consequences	Supp Table 4
**Discussion**			
Limitations	20	Pilot trial limitations, addressing sources of potential bias and remaining uncertainty about feasibility	16
Generalisability	21	Generalisability (applicability) of pilot trial methods and findings to future definitive trial and other studies	16
Interpretation	22	Interpretation consistent with pilot trial objectives and findings, balancing potential benefits and harms, and considering other relevant evidence	13,14,16,17
	22a	Implications for progression from pilot to future definitive trial, including any proposed amendments	13,17
**Other information**			
Registration	23	Registration number for pilot trial and name of trial registry	5
Protocol	24	Where the pilot trial protocol can be accessed, if available	5
Funding	25	Sources of funding and other support (such as supply of drugs), role of funders	18
	26	Ethical approval or approval by research review committee, confirmed with reference number	5

Citation: Eldridge SM, Chan CL, Campbell MJ, Bond CM, Hopewell S, Thabane L, et al. CONSORT 2010 statement: extension to randomised pilot and feasibility trials. BMJ. 2016;355. This is an Open Access article distributed in accordance with the terms of the Creative Commons Attribution (CC BY 3.0) license (http://creativecommons.org/licenses/by/3.0/), which permits others to distribute, remix, adapt and build upon this work, for commercial use, provided the original work is properly cited.

*We strongly recommend reading this statement in conjunction with the CONSORT 2010, extension to randomised pilot and feasibility trials, Explanation and Elaboration for important clarifications on all the items. If relevant, we also recommend reading CONSORT extensions for cluster randomised trials, non-inferiority and equivalence trials, non-pharmacological treatments, herbal interventions, and pragmatic trials. Additional extensions are forthcoming: for those and for up-to-date references relevant to this checklist, see www.consort-statement.org.
